# Within-person Relations between Domains of Socio-emotional Development during Childhood and Adolescence

**DOI:** 10.1007/s10802-022-00933-1

**Published:** 2022-06-07

**Authors:** Lydia Gabriela Speyer, Hildigunnur Anna Hall, Anastasia Ushakova, Michelle Luciano, Bonnie Auyeung, Aja Louise Murray

**Affiliations:** 1 Department of Psychology, University of Edinburgh, Edinburgh, UK; 2 Department of Psychology, University of Cambridge, Downing Site, Cambridge, CB2 3EA UK; 3 Medical School, University of Lancaster, Lancashire, UK; 4 Autism Research Centre, Department of Psychiatry, University of Cambridge, Cambridge, UK

**Keywords:** Developmental psychopathology, Adolescence, Socio-emotional strengths and difficulties, Graphical Vector Autoregression, ALSPAC

## Abstract

Adolescence is a critical period in the development of mental health with nearly 1 in 5 adolescents suffering from mental health problems and more than 40 percent of these experiencing at least one co-occurring mental health disorder. This study investigates whether there are differences in the relations between key dimensions of child and adolescent mental health in adolescence compared to childhood. Mental health and related socio-emotional traits were measured longitudinally at ages 4, 7, 8, 9, 11, 13, and 16 in the Avon Longitudinal Study of Parents and Children (*N* = 11279) using the Strengths and Difficulties Questionnaires. Graphical Vector Autoregression models were used to analyse the temporal within-person relations between conduct problems, emotional problems, hyperactivity/inattention, peer problems and prosociality across childhood (ages 4 to 9) and adolescence (11 to 16). Results suggest that adolescence is characterised by an increase in the number and strength of temporal relations between socio-emotional difficulties. In particular, in adolescence there were bidirectional connections between peer problems and emotional problems, between conduct problems and hyperactivity/inattention and between prosociality and conduct problems as well as hyperactivity/inattention. In childhood, conduct problems and prosociality were reciprocally related. Results also suggested peer problems as a potential mediating factor between conduct and emotional problems in childhood. Overall, this study suggests that different domains of socio-emotional development influence each other over development. Adolescence is characterised by an increase in temporal connections, which may be one factor underlying the increased vulnerability to the onset of mental health problems during that period.

**Supplementary Information:**

The online version contains supplementary material available at 10.1007/s10802-022-00933-1.

Adolescence is a key period in the development of mental health with nearly 1 in 5 adolescents suffering from a mental health disorder (Polanczyk et al., 2015) and more than half of all lifetime reported cases of mental disorders having their onset during this period (Kessler et al., 2005). In addition to an estimated 10–20% of young people suffering from mental health problems severe enough to meet diagnostic criteria, another large proportion is affected by subclinical mental health problems. For instance, a recent study of over 2000 adolescents found that while 17.7% of young people met criteria for a major depressive episode, another 16.5% suffered from subthreshold depression (Crockett et al., 2020). Subclinical levels of ADHD symptoms have also been estimated to affect a much larger proportion of children than those who meet diagnostic criteria (23% vs 3.4% of children worldwide; Balázs & Keresztény, 2014; Polanczyk et al., 2015). Importantly, nearly half of all adolescents with a lifetime mental disorder are estimated to suffer from a co-occurring mental health disorder (Wagner et al., 2017). This not only adds significant complexity to diagnosis and interventions, but also increases the risk of poorer outcomes such as delinquency and unemployment (Sibley et al., 2011).

Over the past decade, an increasing number of studies have not only highlighted the high prevalence of co-occurring mental health disorders, but also the substantial overlap between symptoms of mental health problems (Borsboom, 2017), suggesting that the traditional taxonomic nosology of mental health problems does not adequately capture the complexity of mental health problems. These observations have led to the development of the network approach to psychopathology. Rather than viewing mental health disorders as distinct categories defined by specific impairments caused by an underlying abnormality, the network approach conceptualises disorders as networks of distinct symptoms that interact with each other (Jordan et al., 2020; Robinaugh et al., 2020). In the context of mental health interventions, this approach implies a need to target specific mental health problems and their causal relations to prevent the development of co-occurring disorders.

Focusing on the causal relations between different mental health problems, a large body of research has attempted to understand the development of co-occurring mental health problems within a developmental cascade model framework. According to developmental cascade theories, co-occurring mental health issues are partly the result of longitudinal transactions between different mental health problems and associated behaviours that do not necessarily share a common cause but rather have causal effects on each other (Masten & Cicchetti, 2010). For instance, the dual failure model hypothesises that internalising problems can be the result of cascade effects from externalising problems to problems with peers or academic underachievement that in turn reduce individuals’ self-esteem and consequently lead to internalising problems (Capaldi, 1992). The acting out model, hypothesises cascades from internalising to externalising problems whereby children ‘act out’ to express their distress (Carlson & Cantwell, 1980). Similarly, the ontogenic process model suggests that later mental health problems, and particularly externalising behaviours, are the result of complex longitudinal interactions of individual risk factors, such as heritable trait impulsivity (characteristic of ADHD, for example), with contextual risk factors, such as contact with deviant peers (Beauchaine & McNulty, 2013). While such processes have been investigated in many studies, most of them have only focused on pairwise co-occurring mental health problems, for instance investigating cascades between internalising and externalising problems (e.g. Murray et al., 2020c; van Lier et al., 2012), between externalising and ADHD symptoms (e.g. Kuja-Halkola et al., 2015; Lahey et al., 2002), or between ADHD symptoms and internalising problems (e.g. Murray et al., 2020a; Roy et al., 2014).

Recent work in the Millennium Cohort Study, a large UK-wide longitudinal birth cohort study, has moved beyond the analysis of pairwise comorbidities. Following the network approach to psychopathology, studies have focussed on the developmental interplay between five key mental health domains: conduct problems, emotional problems, hyperactivity/inattention, peer problems and prosociality (Deserno et al., 2021; Speyer et al., 2020). In particular, Speyer et al. (2020) analysed the longitudinal relations between those mental health domains across ages 3, 5, 7, 11, 14, and 17 in the form of longitudinal networks using a multilevel graphical vector autoregression (GVAR) model. Compared to traditional models used to analyse developmental cascades, such as the (random-intercept) cross-lagged panel model, GVAR models are well suited to analysing the inter-relations between a large number variables within one model. It thus helps to operationalise more comprehensive cascade models involving a large number of domains rather than the two or three that are traditionally studied together. Further, GVAR models allow for the disaggregation of within- and between-person effects which is vital in order to isolate the within-person effects referred to in developmental cascade models that are likely to be of primary interest to interventions (Speyer et al., 2020). For instance, interventions targeting peer problems in order to reduce the co-occurrence of conduct problems and emotional problems would only be of benefit if decreases in peer problems in an individual with conduct problems are associate with a subsequent decrease in the same individual’s emotional problems. If associations between conduct problems, peer problems and emotional problems are reflective of between-person differences (i.e. a common dependence on stable factors differing between individuals such as time-stable genetic effects), then interventions targeting peer problems as a means to reducing the co-occurrence of conduct and emotional problems are unlikely to be effective. Thus, if within- and between-person effects are not appropriately disentangled, between-person effects act as a confound for within-person effect resulting in a misleading picture of which intervention targets are likely to be beneficial (Hamaker et al., 2015).

While Speyer et al. (2020) also reported analyses that allowed for some insights into developmental differences in the relations between different mental health domains across time, finding little evidence for differences, the focus was on identifying general patterns over the whole included age range. Those patterns suggested comparably strong reciprocal relations between peer problems and emotional problems, as well as between prosociality and conduct problems. The findings further showed that all of the studied mental health domains (ADHD symptoms, conduct problems, peer problems, emotional problems, and prosociality) influenced each other over time, thus, highlighting the need for more studies to investigate developmental cascades within a more integrated framework that take the complex interplay between multiple mental health domains into account.

Considering that adolescence is a period of increased vulnerability to mental health problems as it is a time of significant physical, psychological and social change (Rapee et al., 2019), it is important to understand if and how mental health relations (i.e. longitudinal associations between different mental health issues) differ from mental health relations in childhood. Prior research has suggested that some dimensions of mental health are likely to become more prominent during adolescence whereas others are likely more central earlier during development. Specifically, adolescence is a period marked by a significant increase in complex social relationships outside the family with peers becoming increasingly important. Consequently, adolescents are more sensitive to peer rejection and approval than younger children, with peer problems likely playing a particularly crucial role in the exacerbation of mental health problems during this developmental period (Rapee et al., 2019). Relatedly, emotional problems have been found to significantly increase during adolescence. This has been suggested to be related to hormonal and morphological changes linked to puberty, which are associated with alterations in sleeping patters, emotion regulation and self-concept, as well as to the greater volatility and complexity of peer relationships (Rapee et al., 2019). Hyperactivity, on the other hand, tends to lead to disruptions in children’s behaviour during the preschool and early school years but then starts to decline (Döpfner et al., 2015) and may thus play a less important role during adolescence. In addition, some mental health problems likely change their manifestation over development. For instance, with regards to conduct problems, aggressive behaviours involving physical contact such as hitting or biting are more common during early childhood. However, as children’s self-regulation and social-information processing abilities increase, indirect forms of aggression, such as social exclusion, tend to become more prominent (Girard et al., 2019). In order to ensure that interventions are developmentally optimal, it is thus vital to know whether key domains of mental health show differential longitudinal interrelations in childhood and adolescence. If different mental health conditions act as antecedents to others in childhood but not in adolescence or vice versa, then there will be different optimal priority targets for the prevention of co-occurring issues in these developmental periods.

The present study adds to Speyer et al.’s (2020) findings by examining the longitudinal relations between key mental health domains as measured by the Strengths and Difficulties Questionnaire (SDQ) within childhood (ages 4, 7, 8, 9 years) and within adolescence (ages 11, 13 and 16 years). In particular, this is the first study to investigate the temporal associations between conduct problems, emotional problems, hyperactivity/inattention, peer problems and prosociality using separate multilevel Graphical Vector Autoregression models (GVAR; Epskamp, 2020) for childhood and adolescence, thus allowing for clearer insights into whether longitudinal mental health relations show developmentally distinct patterns. Data used here comes from the Avon Longitudinal Study of Parents and Children (ALSPAC; Boyd et al., 2013; Fraser et al., 2013), a community-ascertained general population sample from the United Kingdom. While a vast amount of literature has investigated the pairwise co-occurrence of mental health problems in clinical populations;, it is important to complement such studies with community-ascertained samples that can capture the whole spectrum of mental health problems. Such samples further have the benefit of minimising the risk for Berkson’s bias (i.e. overestimation of symptom co-occurrence; Berkson, 1946) and range restriction (i.e. underestimation of symptom co-occurrence; Murray et al., 2014).

## Method

### Participants

The Avon Longitudinal Study of Parents and Children (ALSPAC) is a British longitudinal birth cohort study. Pregnant women resident in Avon, UK with expected dates of delivery from the 1st April 1991 to 31st December 1992 were invited to take part in the study. The initial number of pregnancies enrolled is 14,541 (for these at least one questionnaire has been returned or a “Children in Focus” clinic had been attended by 19/07/99). Of these initial pregnancies, there was a total of 14,676 foetuses, resulting in 14,062 live births and 13,988 children who were alive at 1 year of age (Boyd et al., 2013; Fraser et al., 2013). The study website contains details of available data through a fully searchable data dictionary and variable search tool (http://www.bristol.ac.uk/alspac/researchers/our-data/). ALSPAC is funded by the UK Medical Research Council and Wellcome (Grant ref: 217065/Z/19/Z) and the University of Bristol. Of the participating children, 11,279 had at least one completed SDQ measure and were consequently included in the current study (missing data was dealt with using full information maximum likelihood estimation).

### Ethical Considerations

Ethical approval for the study was obtained from the ALSPAC Ethics and Law Committee and the Local Research Ethics Committees. Informed consent for the use of data collected via questionnaires and clinics was obtained from participants following the recommendations of the ALSPAC Ethics and Law Committee at the time. Further information is available here: http://www.bristol.ac.uk/alspac/researchers/research-ethics.

### Measures

Children’s socio-emotional development was measured using parent-reported Strengths and Difficulties Questionnaires (SDQ; Goodman, 1997) at the children’s following ages: 4, 7, 8, 9, 11, 13, 16. The SDQ is one of the most widely used omnibus measures of mental health problems in general population samples of young people. The SDQ has been validated for ages 4 to 16 and measures children’s psychosocial functioning in five key domains: hyperactivity/inattention, conduct problems, peer problems, emotional problems and prosociality. While not a diagnostic instrument, the SDQ has been found to have good predictive validity for mental disorders, including specific clinical diagnoses for conduct disorders, ADHD and emotional disorders (Goodman et al., 2000). Further, the SDQ has been suggested to be a useful screening tool for identifying mental health problems in primary care settings (Brown & Wissow, 2010) and to monitor outcomes of children at risk for the development of emotional and behavioural issues (Bartal et al., 2020). Thus, even though the SDQ does not measure all mental health problems listed in current diagnostic manuals such as the DSM-V, it is well suited to gain a more comprehensive understanding of how different mental health problems interact longitudinally.

The questionnaire consists of 25 items divided equally between the five different subscales. Items were rated on a three-point scale from ‘not true’ to ‘certainly true’. In the current study, sum-scores of the respective subscale item scores were used. This approach was taken over using a latent measurement model as the high complexity of a latent variable measurement model in conjunction with a GVAR model would have likely led to difficulties with estimation. Higher scores on any of the subscales indicates more behavioural problems, except for the prosociality subscale where higher scores indicate behavioural strengths. The SDQ has been shown to have good predictive validity for diagnoses of mental health disorders in community samples (Goodman et al., 2000) and shows structural, discriminative and convergent validity (Kersten et al., 2016). While analyses of invariance in a different UK birth cohort (The Millennium Cohort Study) indicated that the SDQ shows configural, metric and scalar gender and longitudinal invariance across ages 5 to 14 (Murray et al., 2021b), longitudinal invariance testing in the current sample indicated that configural invariance was not well supported following suggested cut-offs for CFI (observed = 0.88, suggested cut-off > 0.90) and TLI (observed = 0.87, suggested cut-off > 0.90). However, RMSEA (0.061) and SRMR (0.073) were very close to or within acceptable limits (suggested RMSEA cut-off < 0.06, suggested SRMR cut-off < 0.08) (Hu & Bentler, 1999). Prior research has consistently found support for a five-factor model of the SDQ based on RMSEA and SRMR but not based on TLI and CFI (Gomez & Stavropoulos, 2019; Kersten et al., 2016). It has been speculated that this may be due to low average correlations of SDQ items. Specifically, since CFI and TLI values are based on comparing a theoretical model to a null model, i.e. a model with zero correlations between all variables, CFI and TLI values will be low if the theoretical model has low correlations among the variables (i.e. SDQ items). This consequently leads to only a small discrepancy between the theoretical and the null model, resulting in poor CFI and TLI values while RMSEA may still indicate adequate or good fit (Gomez & Stavropoulos, 2019). In such context, it has been suggested that CFI and TLI should not be used as the primary indicator of model fit (Kenny, 2020). Given these findings, we concluded that the limited configural invariance per se is not likely to substantially bias our results. However, longitudinal invariance testing further suggested that metric invariance did not hold and we therefore did not proceed to interpret the results of scalar invariance testing. Thus, when interpreting results of this study, it is important to acknowledge that identified effects may to some extent be confounded by differences in what the SDQ measures at each time-point. Results for configural, metric and scalar invariance are available in the online supplementary materials, Appendix 1. For descriptive statistics, including means, standard deviations, internal consistency (polychoric Omega) and sample sizes at each time point for all included variables, see the online supplementary Table S1.

### Statistical Analyses

To analyse the temporal, contemporaneous and between-person relations between the different subscales of the SDQ, multilevel Graphical Vector Autoregression (GVAR) models were fitted. To be able to investigate whether there are differences in these associations in childhood compared to adolescence, two models were built for ages 4 to 9 (childhood network) and for ages 11 to 16 (adolescence network).

GVAR models are well suited to model temporal processes that can be described through the presence of a serial dependency, also known as autocorrelation. In GVAR models, variables that have been measured repeatedly are modelled as a function of their past values or as a combined function of their past values as well as the past values of other variables. Most commonly, a lag of one is chosen which assumes that the current value of a variable depends on its value at the preceding time point (Epskamp, 2020). GVAR models are mostly used to analyse temporal associations between different variables, but also allow for insights into contemporaneous within-time associations (Wild et al., 2010). Multilevel GVAR models estimate the cross-sectional between-person differences across time, which allows separation of within- and between-person effects as within-person effects are essentially estimated on the residuals after accounting for between-person differences. Thus, the model implicitly controls for stable between-person factors such as stable demographic factors. Structurally, the GVAR model is closely related to the random-intercept cross-lagged panel model (RI-CLPM) which allows for the estimation of within-person effects if at least three observations per participant are available (Hamaker et al., 2015). However, unlike RI-CLPMs, GVAR models can more easily accommodate the inclusion of many variables as they are computationally simpler. A further difference between RI-CLPMs and GVAR models relates to their treatment of the first measurement wave. In contrast to RI-CLPMs, GVAR models do not treat the first wave of measurements as exogenous with a unique set of variances and covariances. Instead, using a Kronecker product structure, GVAR models compute the implied variance–covariance structure of the first measurement wave based on the temporal and contemporaneous network structures as well as the assumption of stationarity, leading to a more parsimonious model (Deserno et al., 2021). Future research should investigate in how far these differences between RI-CLPMs and GVAR models affect empirical results.

GVAR models further model the within-person and between-person covariance structures in the form of Gaussian Graphical Models (GGMs) rather than as marginal variance–covariance matrices (Epskamp, 2020). This offers a clear advantage over RI-CLPMs as GGMs allow for an intuitive visualisation of the complex dependency structures in a system of multiple variables using partial correlations (Epskamp, 2020). Graphical models represent variables as nodes that are connected by edges indicating the relations between the variables. For the temporal GVAR network, these edges include arrows to visualise the direction of effects. In the contemporaneous networks, which are estimated based on the residualised covariance structure after accounting for temporal effects, the edges are undirected, hence, visualising conditional dependencies between variables. The between-person network describes the associations between the stationary means of all subjects also using undirected edges (Epskamp et al., 2018). In the present study, between-person networks were not of primary interest as the cascades hypothesised by developmental psychopathology models refer to within-person processes. Nevertheless, all results are reported for completeness.

In the current study, GVAR models were built using the panelgvar() function from the *psychonetrics* package (Epskamp, 2020). Since GVAR models assume stationary relations across time, prior to fitting the models, data was detrended for linear, quadratic and cubic age-related effects and was then standardised across time points. This was considered appropriate for the presented analyses since we were interested in the correlational structure rather than the mean structure of the included variables and such standardisation does not affect the within-person relations of interest.

To account for missing data, both models were estimated using Full Information Maximum Likelihood which provides unbiased parameter estimates given that data are missing at random (Enders, 2001). To reduce the complexity of the models and to decrease the chance of finding false positives, models were further regularised using Bayesian Information Criterion (BIC) as model selection criterion. To evaluate the fit of the final models, Comparative Fit Index (CFI), Tucker Lewis Index (TLI) and Root Mean Square Error of Approximation (RMSEA) were computed with CFI > 0.90, TLI > 0.90 and RMSEA < 0.05 used as cut off criteria indicating reasonably good fit (Kline, 2005). The R package *qgraph* (Epskamp et al., 2012) was used to visualise the models and 25% case-drop bootstrapping routines (*N* = 1000) were performed to evaluate the stability of parameter estimates (Epskamp, 2020).

Models were further compared using the following descriptive network statistics calculated using the R packages *igraph* (Csárdi & Nepusz, 2006) and *network* (Butts, 2008): Network Density (*ρ*), describing the proportion of edges included in the network out of all possible connections (possible range 0–1, 0 = no edges, 1 = all edges included), the Global Clustering Coefficient (*GCC*), measuring the degree to which nodes in the network cluster together (0 = no two nodes also share an edge with a third node, 1 = all nodes share an edge with a mutual third node), and the Weighted Jaccard Distance (*d*_*J*_*)*, an indicator of the ratio of shared edges in comparison to all edges (0 = no edges overlap, 1 = all edges overlap) (Tantardini et al., 2019).

## Results

**Fig. 1:**
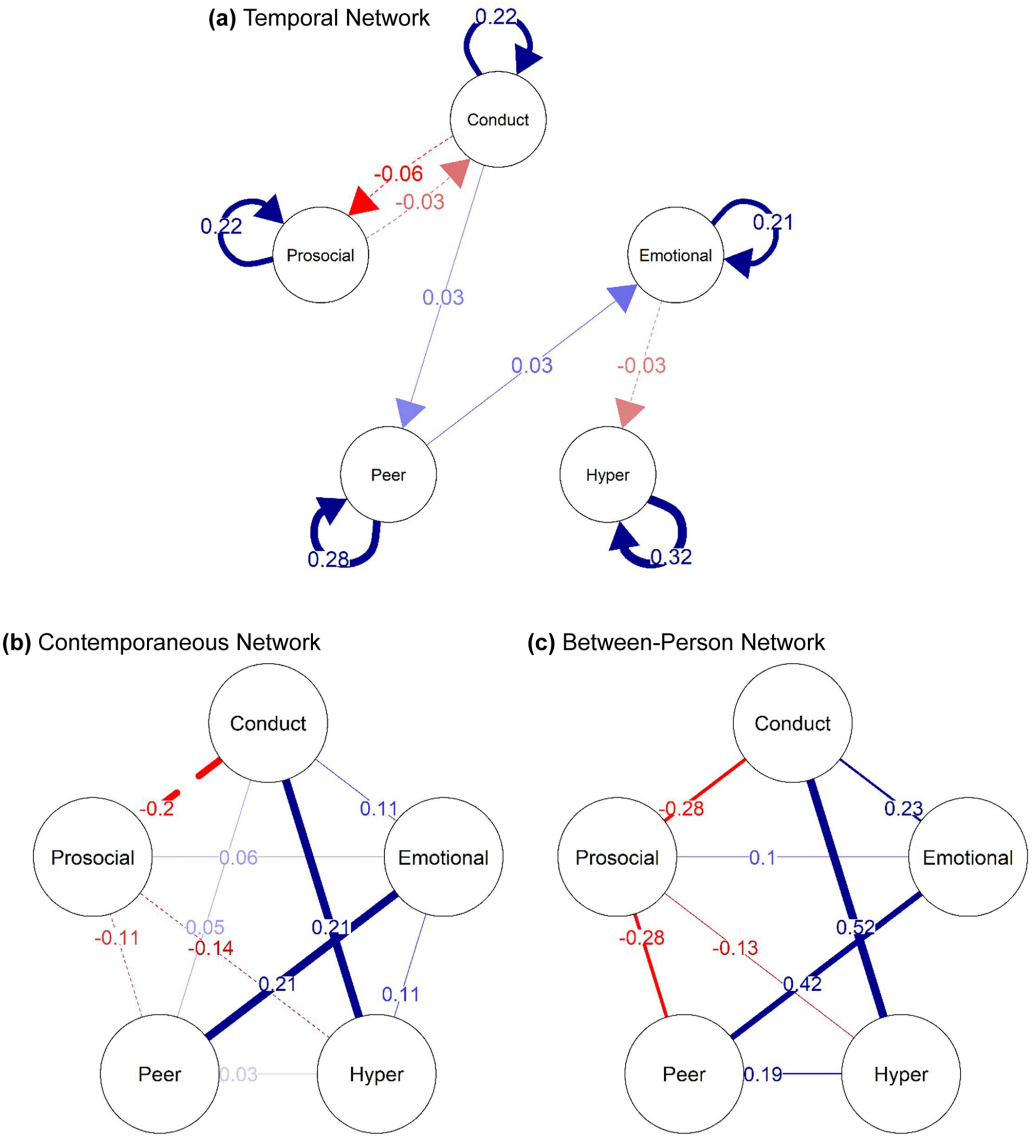
Childhood Networks: (**a**) fixed-effect within-person temporal GVAR network standardised to directed partial correlations, (**b**) fixed-effect within-person contemporaneous partial correlation network, and (**c**) random-effects between-person partial correlation network. Red edges (dashed lines) indicate negative effects; blue edges (solid lines) indicate positive effects

The saturated childhood GVAR model which included all potential edges showed good fit (*CFI* = 0.95, *TLI* = 0.95 and *RMSEA* = 0.041 with 90% *CI*: 0.040 to 0.042). The regularised model performed slightly better than the saturated model (∆BIC = 112.77) and also showed good fit (*CFI* = 0.95, *TLI* = 0.95 and *RMSEA* = 0.039 with 90% *CI*: 0.038 to 0.040). Figure 1 visualises the structures of the temporal (a), contemporaneous (b) and between-person (c) networks. For parameter estimates of the standardised childhood networks, see Table S2 in the online supplementary materials. The temporal childhood network indicated that, at the within-person level, prosocial behaviour and conduct problems show reciprocal negative relations. In addition, it suggested that conduct problems are associated with more peer problems over time which are in turn associated with increases in emotional problems. Emotional problems were further associated with decreases in hyperactivity/inattention. The contemporaneous network suggested that all mental health domains were associated within the same time points. It highlighted that peer problems and emotional problems as well as conduct problems and hyperactivity/inattention shared comparatively strong links. Also, prosociality was associated with lower problems in all domains except emotional problems with which it shared a positive link. Bootstrapping results indicated that the contemporaneous and the between-person network were fairly stable while the temporal network may potentially be less sparse than estimated in the regularised childhood GVAR model (Online Supplementary Table S3).

**Fig. 2:**
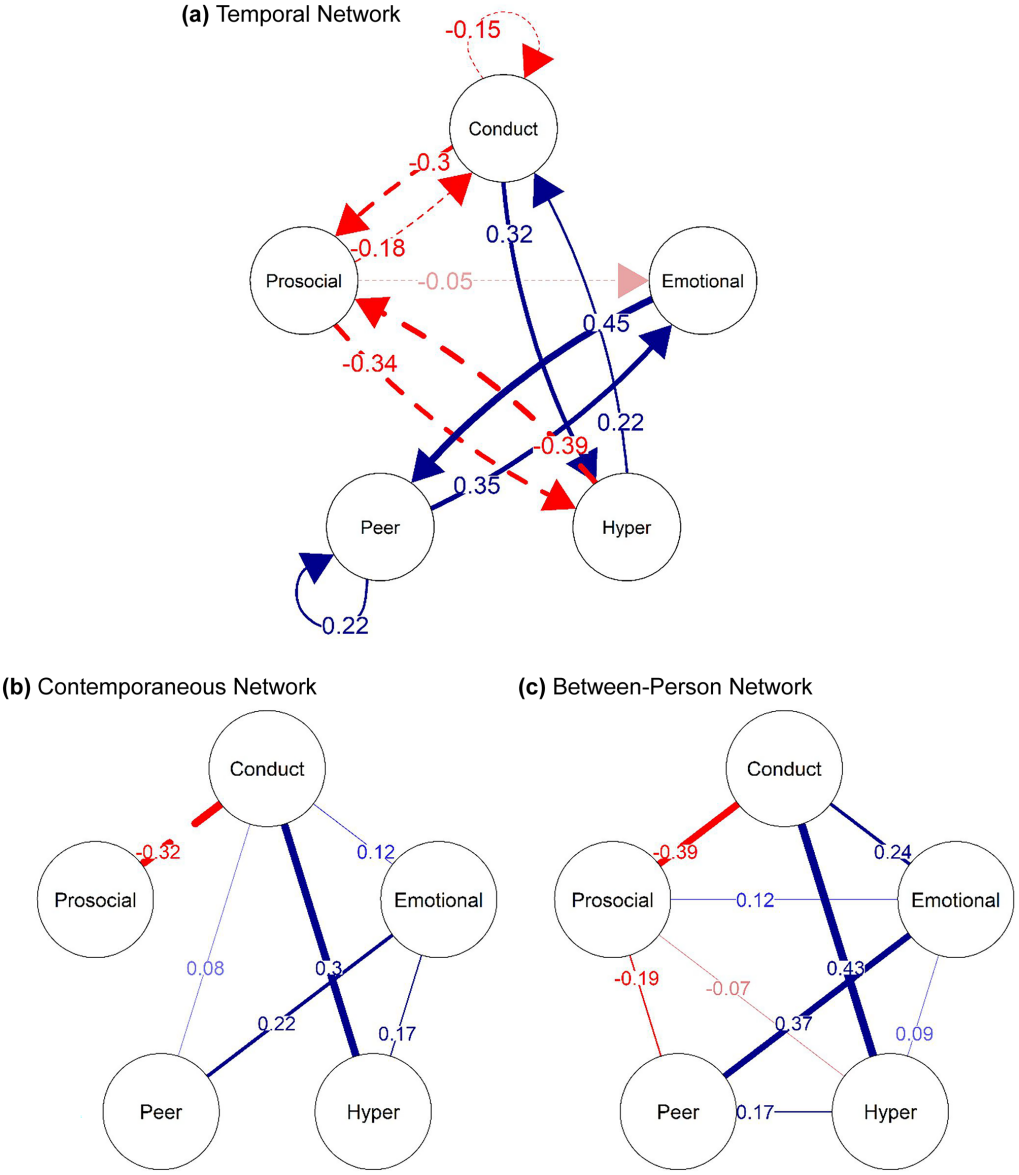
Adolescence Networks: (**a**) fixed-effect within-person temporal GVAR network standardised to directed partial correlations, (**b**) fixed-effect within-person contemporaneous partial correlation network, and (**c**) random-effects between-person partial correlation network. Red edges (dashed lines) indicate negative effects; blue edges (solid lines) indicate positive effects

The saturated adolescence GVAR model showed excellent fit (*CFI* = 0.97, *TLI* = 0.97 and *RMSEA* = 0.031 with 90% *CI*: 0.029 to 0.032) with the regularised model again performing slightly better (∆BIC = 98.55) and also showing excellent fit (*CFI* = 0.97, *TLI* = 0.97 and *RMSEA* = 0.030 with 90% *CI*: 0.028 to 0.031). Temporal (a), contemporaneous (b) and between-person (c) adolescence networks are visualised in Fig. 2. For parameter estimates see Table S4 in the online supplementary. The temporal adolescence network highlighted reciprocal relations between a number of different mental health domains. In particular, prosociality shared bidirectional negative associations with conduct problems, and hyperactivity/inattention; peer problems and emotional problems as well as conduct problems and emotional problems showed bidirectional positive associations. Prosociality further showed a small negative effect on later emotional problems. The contemporaneous network additionally highlighted that emotional problems were associated with more peer problems, conduct problems and hyperactivity/inattention, while conduct problems were also associated with more peer problems and hyperactivity/inattention as well as with lower prosociality. Bootstrapping results for the adolescence GVAR model revealed essentially the same pattern as for the childhood GVAR showing that the between-person and the contemporaneous network were relatively stable while the temporal network may be less sparse than originally estimated (Online Supplementary Table S5).

Visual comparison of the two networks indicated that the temporal adolescence network is considerably more connected than the temporal childhood network. Network density tests confirmed this (*ρ*(childhood) = 0.25; *ρ*(adolescence) = 0.45). The *GCC* for the childhood network indicated that no two nodes were also connected to a third mutual node (*GCC* = 0), whereas nodes in the adolescence network had a high probability of also sharing a connection with a third node (*GCC* = 0.50), thus further indicating that mental health problems were much more interconnected during adolescence than during childhood. Finally, the Weighted Jaccard Distance showed that only around a third of the potential edges overlapped between the two networks (*d*_*J*_ = 0.31). The contemporaneous network and the between-person network seemed to share very similar structures, although the connections in the adolescence networks were slightly stronger than in the childhood network as was confirmed by network density statistics confirmed (available in Table 1).

**Table 1:** Descriptive Network Statistics

		Density	*GCC*	Jaccard Distance
Temporal Network:	Childhood	0.25	0.00	
	Adolescence	0.45	0.50	0.31
Contemporaneous Network:	Childhood	1.00	1.00	
	Adolescence	0.60	0.55	0.60
Between-Person Network:	Childhood	0.80	0.67	
	Adolescence	0.90	0.88	0.89

*GCC =* Global Clustering Coefficient

## Discussion

This study’s aim was to build on previous evidence suggesting widespread developmental connections between different domains of mental health to explore possible differences in the relations between socio-emotional strengths and difficulties in adolescence compared to childhood. We found that whereas concurrent associations between the different SDQ domains were relatively similar in the two networks, cross-domain temporal relations exhibited different patterns. In particular, compared to childhood, the period of adolescence seems to be characterised by an increase in temporal connections between different mental health domains. Overall, within-person effects were rather small; however, considering the longitudinal modelling context, this was to be expected given that within-person effects are estimated after controlling for stability effects. It is important to note that such effects may still be meaningful in a clinical context as they tend to accumulate over time (see Adachi & Willoughby, 2014 for a detailed discussion). Between-person effects, on the other hand, were rather large, suggesting that risk factors that differ between individuals, such as genetics or stable aspects of the family environment play an important role in why socio-emotional difficulties commonly co-occur.

In line with Speyer et al.’s (2020) findings, both the childhood and the adolescence network highlighted negative reciprocal temporal relations between conduct problems and prosociality. While conduct, peer and emotional problems as well as hyperactive/inattentive behaviours have received a substantial amount of attention in the developmental cascade literature, much less attention has been on prosociality. Previous research on children and adolescents has, however, consistently found evidence for negative effects of conduct problems on prosociality. This has sometimes be explained in terms of peer deviancy training whereby aggressive children have fewer opportunities to develop prosocial skills because they are rejected by normative peers and instead gravitate towards antisocial peers (Obsuth et al., 2015). However, findings for the opposite direction, from prosociality to conduct problems, are rare (Chen et al., 2010; Speyer et al., 2020), possibly because conduct problems may be rather stable over time and consequently, this effect is only revealed when partialling out between-person differences. An implication of this is that teaching children the skills to behave prosocially and providing them with opportunities to enact those skills could help in the prevention of conduct problems. Alternatively, it may be that the bidirectional associations between conduct problems and prosociality observed here reflect the strong links between conduct problems and psychopathy. Specifically, one aspect of prosociality, that is limited prosocial emotions, was recently introduced as a specifier of conduct disorder to the DSM-5 and ICD-11 (Colins et al., 2021). Limited prosocial emotions are a key feature of callous-unemotional traits (a precursor or marker of psychopathic traits) which have been linked to difficulties with associating harmful behaviours with negative emotional cues from the environment, leading to children failing to recognise that their behaviour may harm others (Colins et al., 2021). While children with low CU traits would modulate their own behaviours in response to cues from their environment, children with high CU traits are likely to miss out on the modulation of their behaviours, putting them at risk for the development of continuous and severe behavioural issues (Waller et al., 2014). In parts, this difficulty in associating harmful behaviours with negative emotional cues, may stem from the fact that children with high CU traits likely require increased levels of emotional arousal. Normally, parental socialisation efforts produce the required negative emotional arousal that enable the development of conscience and the internalisation of norms. However, if a child does not achieve optimal arousal levels, for instance due to amygdala hypoactivity, the development of conscience will not take place as expected. This may subsequently result in the development of aggressive behaviours as societal norms are not adequately recognised (Waller et al., 2014). The literature on such processes however suggests that these processes are primarily at play during early childhood with CU traits being relatively stable during adolescence (Pisano et al., 2017). This makes it a more likely mechanism for associations between prosociality and conduct problems in childhood rather than adolescence.

Results of the childhood network further suggested that, over time, conduct problems are associated with increases in peer problems and peer problems are associated with increases in emotional problems. There was no link between conduct problems and emotional problems, suggesting that these two domains are only indirectly linked via peer problems. This is consistent with the existing literature on conduct problems and emotional difficulties in childhood which has found that their associations are mediated by peer problems as hypothesised by the dual failure model (Gooren et al., 2011; van Lier & Koot, 2010; van Lier et al., 2012). However, in contrast to the childhood network, the adolescence network indicated that conduct problems are not associated with either increases in peer problems or emotional problems which suggests that the mechanisms implied by the dual failure model may be more prominent in childhood. In adolescence, conduct problems are generally considered more normative as is also evident in a delinquency spike during this developmental period (Murray et al., 2021a). Thus, conduct problems may not necessarily be associated with peer problems during this period as would be hypothesised by the dual failure model. This is also in line with previous research that has found that evidence for the social pathway of the dual failure model is less consistent at later ages (Boutin et al., 2020) with academic performance potentially playing a stronger role in mediating the associations between externalising and internalising difficulties (Obradović et al., 2010). Ideally, future studies should extend the current study by additionally incorporating academic achievement into their analyses to investigate the role of both academic performance and peer problems in the relations between conduct problems and emotional problems in a more confirmatory framework that also allows for the testing of mediation effects.

The childhood network also suggested that, over time, higher emotional problems are linked to lower hyperactivity/inattention. The temporal adolescence network, however, showed no such effect. This hints at a potential protective effect of emotional problems against hyperactive/inattentive behaviours during early development. To date, only a handful of studies have found evidence for effects of emotional problems on ADHD symptoms with evidence being mixed. Some studies found that emotional problems protected against later ADHD symptoms (Obsuth et al., 2020) while others found that emotional problems were associated with increases in ADHD symptoms in adolescence (Murray et al., 2020a). One possible explanation for a protective effect of emotional problems against ADHD symptoms could be that children with emotional difficulties might be more fearful of consequences resulting from hyperactive/inattentive behaviour and hence more inhibited. This is in line with the attenuation hypothesis which proposes that the behavioural inhibitions associated with anxiety counteract the overt symptoms of ADHD (Murray et al., 2018). Considering that ADHD is highly heritable (Larsson et al., 2014), and associated with poorer abilities in detecting social cues (Parke et al., 2018), it may be that parents with ADHD traits themselves might miss the subtle signs of emotional problems in children with ADHD. That this protective effect was only evident in childhood suggests that the mechanisms underlying the observed protective role of emotional problems are no longer at play during adolescence.

During adolescence, ADHD symptoms further showed reciprocal relations with conduct problems as well as prosociality. While a large amount of literature has found that ADHD symptoms are a strong risk factor for later conduct problems as, for example, hypothesised by the ontogenic process model (Beauchaine & McNulty, 2013; Beauchaine et al., 2010), prosociality has received comparably little attention in studies investigating the links between multiple different mental health problems. In fact, despite the evidence for its links with issues such as conduct problems, prosociality does not feature prominently in developmental cascade theories such as the dual failure model (Capaldi, 1992). This may partly be due to the fact that, in the literature, prosociality has predominantly been considered in the context of psychopathy. However, in line with Speyer et al.’s (2020) study, we found evidence for within-person associations of prosociality with all other domains of socio-emotional development, highlighting that its incorporation into developmental cascade models has the potential to improve their explanatory power. It also suggests that interventions aiming to promote prosocial behaviours may be beneficial in reducing socio-emotional problems and may help prevent the development of co-occurring mental health problems. Future studies are needed to gain a better understanding of how prosociality becomes linked to other socio-emotional difficulties.

Finally, the adolescence network also showed evidence for temporal relations between peer problems and emotional problems. While in the childhood network, these were unidirectional with peer problems leading to increases in emotional problems but not vice versa, during adolescence, emotional problems were also associated with changes in peer problems. Previous research already reports such bidirectional effects (e.g., Speyer et al., 2020), however, most studies have focused on middle childhood allowing only limited insights into developmental differences (Reijntjes et al., 2010). As children grow up, they spend increasingly more time with their peers, with peer relationships becoming more complex. The increased salience of peers during this period likely amplifies the effect of negative peer interactions (e.g. rejection, victimisation) on children’s emotional wellbeing and vice versa (Rapee et al., 2019). One mechanism underlying the association of emotional problems with peer problems could be related to the fact that internalising issues such as anxiety or depression lead to increased irritability (Humphreys et al., 2019). Irritability, in turn may lead to an escalation of peer conflicts, resulting in a vicious cycle whereby peer problems reinforce emotional issues that make it harder for individuals to cope with the demands of increasingly complex peer relationships (Hames et al., 2013; Zhang et al., 2020).

While not the primary focus of the analyses presented in the current manuscript, it is important to note that the between-person networks suggested that all studied mental health domains were connected fairly strongly during childhood as well as adolescence, with these interconnections being comparably stronger than the observed longitudinal within-person effects. The strong between-person effects observed here highlight that stable factors differing between individuals also play a significant role in the co-occurrence of mental health problems. These findings are in line with previous research highlighting that most mental health problems share common aetiological factors that mostly reflect between-person differences (Caspi & Moffitt, 2018). For instance, polygenic risk scores for ADHD have been found to be associated with both internalising and externalising difficulties, as have a variety of other risk factors such as maternal history of depression or smoking during pregnancy (Murray et al., 2020b; Speyer et al., 2021). With regards to interventions, this suggests that in addition to focusing on targeting pathways that potentially increase the likelihood of someone suffering from one mental health problem to develop another mental health problem, it is highly important to identify those individuals most at risk for developing co-occurring mental health problems based on the broader set of risk factors an individual presents with. Transdiagnostic interventions that target a range of difficulties rather than only a subset of symptoms may consequently be particularly beneficial in reducing the co-occurrence of mental health problems in individuals exposed to risk factors that are associated with a variety of mental health problems (Dalgleish et al., 2020; Kotov et al., 2021). Future studies that integrate between-person predictors into the analysis of within-person relations may be beneficial to guide such interventions.

### Limitations

The main limitation of the current study is that it relied on an omnibus measure of mental health that, while recommended for use in clinical settings, was not designed to diagnose specific mental health conditions. Thus, findings of the current study only allow insights into longitudinal associations between different, fairly broad, mental health domains but not for insights into associations between specific disorders. Neither did this study allow for the explicit testing of developmental psychopathology theories such as the dual-failure model, as the necessary mediating factors were not available comparably over the different waves. Future research should test these theories within a more confirmatory framework that also allows for mediation and moderation analyses. Also, the SDQ was designed to capture socio-emotional strengths and difficulties in a large age group (4 to 16), thus, it focuses primarily on symptoms that are relevant across different developmental stages. For example, the measure on peer problems is quite general, allowing for insights into peer problems both during childhood and during adolescence. However, peer interactions increase in complexity and importance during adolescence, as well as occurring in different contexts but this is not reflected in any changes in the item contents. Future studies, exploring differences in longitudinal associations between a range of mental health conditions, ideally using measures that can adequately capture different symptom presentations at different ages, would consequently be highly valuable. Further, the current study relied exclusively on parental reports. This might be problematic, especially for the later time points, as parental monitoring and knowledge likely declines in adolescence, making parent-reports less reliable (Masche, 2010). Future studies should replicate this study using self- and teacher-reports alongside parent-reports. Considering that the GVAR models and the corresponding descriptive network comparisons between the two models were exploratory due to the lack of formal network comparison tests for longitudinal networks available as of yet, replication in independent data and confirmatory modelling strategies are needed. Also, GVAR models assume stationary relations across time, thus, making it impossible to investigate whether there are more gradual changes in magnitude of effects across development. Future studies, for instance using continuous time modelling, are necessary to investigate whether there are more gradual changes in the longitudinal relations between different domains of socio-emotional development. Another limitation to consider is the non-normal distribution of some of the included variables. This could have affected our estimates in a hard to predict direction given that temporal networks may be sensitive to violation of the normality assumption (Epskamp et al., 2018). Also, invariance analyses only found limited support for configural invariance of the SDQ and suggested that metric and scalar invariance did not hold in the ALSPAC data, thus, our longitudinal effects may to some extent be confounded by measurement differences in the SDQ across development. Finally, a study on attrition in ALSPAC found that children with behavioral difficulties were more likely to drop-out (Wolke et al., 2009), hence, this could have led to underestimated effect sizes. However, using simulations, the same study also found that this selective drop-out only had marginal effects on the validity of analyses conducted using the ALSPAC cohort.

## Conclusion

Overall, the findings of this study emphasise that children and adolescents’ strengths and difficulties in different socio-emotional domains influence each other over- and within-time. Compared to childhood, adolescence is characterised by an increase in temporal connections, likely reflecting the increased vulnerability to the onset of mental health problems during that period. Results further highlight that all domains of socio-emotional development are connected to at least one other domain. Prosociality featured prominently, emphasising the clear need for more encompassing theories of developmental psychopathology that includes this important psychosocial factor. More broadly, our results highlight the need for more comprehensive but also developmentally tailored cascade theories that acknowledge the developmentally varying inter-connectedness of psychosocial issues across a wide range of domains. This can also help inform interventions to target the symptoms that are most likely to lead to the development of others during specific periods throughout children’s and adolescents’ development.

## Supplementary Information

Below is the link to the electronic supplementary material.

Supplementary file1 (DOCX 57 KB)

## Data Availability

ALSPAC data access is through a system of managed open access. For details, see http://www.bristol.ac.uk/alspac/researchers/access/.
