# Supplementary material for: Within-person Relations between Domains of Socio-emotional Development during Childhood and Adolescence
Source: Res Child Adolesc Psychopathol. 2022 Jun 7;50(10):1261–74. doi: 10.1007/s10802-022-00933-1 (PMC9606067; doi:10.1007/s10802-022-00933-1)
Supplement: Supplementary file 1 — Supplementary file1 (DOCX 57 KB) [file 10802_2022_933_MOESM1_ESM.docx]

#### Supplementary Materials

**Within-Person Relations between Domains of Socio-Emotional Development during Childhood and Adolescence**

**Appendix 1. Longitudinal Invariance Testing**

| **Table A1.** Model fit for longitudinal invariance model | | | | | | |
| --- | --- | --- | --- | --- | --- | --- |
| **Model** | **CFI** | **ΔCFI** | **RMSEA** | **ΔRMSEA** | **SRMR** | **ΔSRMR** |
| Configural | .884 | - | .061 | - | .073 | - |
| Metric | .872 | .012 | .062 | .001 | .076 | .003 |
| Metric and Scalar | .868 | .004 | .061 | -.001 | .077 | .001 |
| *Note.* CFI = Comparative Fit Index; TLI = Tucker Lewis Index; RMSEA = Root Mean Square Error of Approximation; SRMR = Standardised Root Mean Square Residual; To judge whether metric or configural invariance held, we followed the guidelines proposed by Chen (2007). | | | | | | |

Longitudinal invariance was tested within a Confirmatory Factor Analysis (CFA) Framework (for details see Liu et al., 2017). In particular, we first tested a configural model following the SDQ’s hypothesised five-factor structure. The pattern of factor loadings were fixed to be equal across time but magnitude of factor loadings and thresholds were allowed to vary. Model fit of the configural model was judged based on Comparative Fit Index (CFI) >.90, Tucker Lewis Index (TLI) >.90, Root Mean Squared Error of Approximation (RMSEA) <.06 and Standardised Root Mean Square Residual (SRMR) <.08 (Hu & Bentler, 1999). If model fit for the configural model was judged to be adequate, we tested for metric invariance by adding cross-time-point equality constraints for factor loadings. Following the guidelines put forward by Chen (2007), we judged metric invariance to hold if, compared to the configural model, CFI values decreased no more than by .010, RMSEA increased by no more than .015 SRMR increased by no more than .030. If metric invariance held, we added equality constraints across time for item thresholds to test for scalar invariance. To judge scalar invariance, we used the same criteria as testing for metric invariance (except using increases of .010 as cut-off for SRMR), comparing the fit of the metric invariant model to the model with scalar invariant constraints imposed (Chen, 2007). Analyses were implemented using the ‘convenience features’ for invariance testing available in Mplus 8.7 (Muthén & Muthén, 2018). For details regarding constraints imposed for model identification, see Version 7.1 Mplus Language Addendum available at <http://www.statmodel.com/>).

Model fit indices for the configural, metric and scalar invariance model are listed in Table A1. These suggested only limited support for the configural model and further indicated that metric invariance did not hold as CFI decreased by more than .01 after introducing cross-time-point equality constraints on factor loadings. Full results for factor loadings and thresholds for all reported models are available on the Open Science Framework: <https://osf.io/3hsym/>

**References**

Chen, F. F. (2007). Sensitivity of goodness of fit indexes to lack of measurement invariance. *Structural Equation Modeling*, *14*(3), 464–504. https://doi.org/10.1080/10705510701301834

Hu, L. T., & Bentler, P. M. (1999). Cutoff criteria for fit indexes in covariance structure analysis: Conventional criteria versus new alternatives. *Structural Equation Modeling*, *6*(1), 1–55. https://doi.org/10.1080/10705519909540118

Liu, Y., Millsap, R. E., West, S. G., Tein, J. Y., Tanaka, R., & Grimm, K. J. (2017). Testing measurement invariance in longitudinal data with ordered-categorical measures. *Psychological Methods*, *22*(3), 486–506. https://doi.org/10.1037/MET0000075

Muthén, L. K., & Muthén, B. (2018). Mplus. *The Comprehensive Modelling Program for Applied Researchers: User’s Guide*, *5*.

| **Table S1.** Descriptive statistics | | | | | | | | |  |
| --- | --- | --- | --- | --- | --- | --- | --- | --- | --- |
|  | ***N*** | ***Mean*** | ***SD*** | ***Min*** | ***Max*** | ***Skew*** | ***Kurtosis*** | ***ω*** |  |
| Age 4 Conduct Problems | 9374 | 1.958 | 1.409 | 0 | 10 | 0.786 | 0.882 | .77 |  |
| Age 4 Emotional Problems | 9383 | 1.450 | 1.511 | 0 | 10 | 1.256 | 1.659 | .79 |  |
| Age 4 Hyperactivity | 9293 | 3.967 | 2.324 | 0 | 10 | 0.442 | -0.271 | .89 |  |
| Age 4 Peer Problems | 9317 | 1.524 | 1.483 | 0 | 9 | 1.032 | 0.935 | .70 |  |
| Age 4 Prosociality | 9352 | 7.050 | 1.980 | 0 | 10 | -0.341 | -0.336 | .84 |  |
| Age 7 Conduct Problems | 8175 | 1.572 | 1.439 | 0 | 10 | 1.022 | 1.206 | .81 |  |
| Age 7 Emotional Problems | 8197 | 1.492 | 1.658 | 0 | 10 | 1.328 | 1.674 | .82 |  |
| Age 7 Hyperactivity | 7997 | 3.379 | 2.372 | 0 | 10 | 0.636 | -0.135 | .90 |  |
| Age 7 Peer Problems | 7749 | 1.021 | 1.381 | 0 | 10 | 1.798 | 3.978 | .81 |  |
| Age 7 Prosociality | 8175 | 8.200 | 1.732 | 0 | 10 | -0.848 | 0.142 | .87 |  |
| Age 8 Conduct Problems | 7674 | 1.492 | 1.459 | 0 | 10 | 1.232 | 2.149 | .86 |  |
| Age 8 Emotional Problems | 7693 | 1.679 | 1.825 | 0 | 10 | 1.230 | 1.266 | .85 |  |
| Age 8 Hyperactivity | 7665 | 3.339 | 2.458 | 0 | 10 | 0.664 | -0.094 | .92 |  |
| Age 8 Peer Problems | 7610 | 1.301 | 1.534 | 0 | 10 | 1.512 | 2.677 | .81 |  |
| Age 8 Prosociality | 7693 | 8.023 | 1.893 | 0 | 10 | -0.755 | -0.215 | .88 |  |
| Age 9 Conduct Problems | 7619 | 1.233 | 1.370 | 0 | 10 | 1.458 | 2.881 | .85 |  |
| Age 9 Emotional Problems | 7697 | 1.486 | 1.743 | 0 | 10 | 1.490 | 2.344 | .86 |  |
| Age 9 Hyperactivity | 7600 | 2.917 | 2.245 | 0 | 10 | 0.834 | 0.333 | .90 |  |
| Age 9 Peer Problems | 7414 | 1.075 | 1.455 | 0 | 10 | 1.814 | 3.867 | .84 |  |
| Age 9 Prosociality | 7735 | 8.350 | 1.636 | 0 | 10 | -1.016 | 0.766 | .85 |  |
| Age 11 Conduct Problems | 6981 | 1.155 | 1.367 | 0 | 10 | 1.571 | 3.275 | .87 |  |
| Age 11 Emotional Problems | 7027 | 1.437 | 1.709 | 0 | 10 | 1.528 | 2.503 | .85 |  |
| Age 11 Hyperactivity | 6971 | 2.746 | 2.226 | 0 | 10 | 0.939 | 0.612 | .91 |  |
| Age 11 Peer Problems | 6752 | 1.044 | 1.489 | 0 | 9 | 1.885 | 3.989 | .85 |  |
| Age 11 Prosociality | 7014 | 8.364 | 1.664 | 0 | 10 | -1.049 | 0.800 | .85 |  |
| Age 13 Conduct Problems | 6758 | 1.199 | 1.365 | 0 | 10 | 1.506 | 3.095 | .87 |  |
| Age 13 Emotional Problems | 6864 | 1.401 | 1.686 | 0 | 10 | 1.586 | 2.764 | .86 |  |
| Age 13 Hyperactivity | 6825 | 2.904 | 2.218 | 0 | 10 | 0.791 | 0.289 | .91 |  |
| Age 13 Peer Problems | 6519 | 1.151 | 1.568 | 0 | 10 | 1.855 | 3.933 | .85 |  |
| Age 13 Prosociality | 6773 | 8.242 | 1.716 | 1 | 10 | -1.000 | 0.513 | .87 |  |
| Age 16 Conduct Problems | 5319 | 0.944 | 1.239 | 0 | 9 | 1.859 | 4.782 | .87 |  |
| Age 16 Emotional Problems | 5371 | 1.449 | 1.822 | 0 | 10 | 1.685 | 3.125 | .89 |  |
| Age 16 Hyperactivity | 5309 | 2.518 | 2.099 | 0 | 10 | 0.951 | 0.701 | .90 |  |
| Age 16 Peer Problems | 5065 | 1.020 | 1.386 | 0 | 10 | 1.915 | 4.726 | .84 |  |
| Age 16 Prosociality | 5113 | 8.053 | 1.822 | 0 | 10 | -0.966 | 0.488 | .87 |  |
| *Note. ω =* McDonalds Omega using polychoric correlations (internal consistency). | | | | | | | | |  |

| **Table S2**: Estimates for regularised Childhood GVAR Model  **(a) Temporal Relations** | | | | | | | | | | | | | | | |
| --- | --- | --- | --- | --- | --- | --- | --- | --- | --- | --- | --- | --- | --- | --- | --- |
|  | | | a. | | | b. | | | c. | | | d. | | | e. |
| 1. Conduct Problems (a) | | | 0.220 | | | - | | | - | | | 0.029 | | | -0.061 |
| 2. Emotional Problems (b) | | | - | | | 0.210 | | | -0.03 | | | - | | | - |
| 3. Hyperactivity (c) | | | - | | | - | | | 0.317 | | | - | | | - |
| 4. Peer Problems (d) | | | - | | | 0.034 | | | - | | | 0.278 | | | - |
| 5. Prosociality (e) | | | -0.033 | | | - | | | - | | | - | | | 0.219 |
| *Note:* Numeric estimates represent estimated partial directed correlation, diagonal elements represent autocorrelations, lower triangle represents directional relations from 1-5 to a-e and the upper triangle directional relations from a-e to 1-5 (e.g., -0.061 = conduct problems (1) 🡪 prosociality (e); -0.033 = prosociality (5) 🡪 conduct problems (a)). | | | | | | | | | | | | | | | |
| **(b) Contemporaneous Relations** | | | | | | | | | | | | | | | |
|  | 1. | | | | 2. | | | 3. | | | 4. | | | 5. | |
| 1. Conduct Problems | - | | | | 0.158 | | | 0.263 | | | 0.124 | | | -0.243 | |
| 2. Emotional Problems | 0.114 | | | | - | | | 0.152 | | | 0.224 | | | -0.023 | |
| 3. Hyperactivity | 0.205 | | | | 0.111 | | | - | | | 0.100 | | | -0.197 | |
| 4. Peer Problems | 0.054 | | | | 0.207 | | | 0.031 | | | - | | | -0.138 | |
| 5. Prosociality | -0.197 | | | | 0.056 | | | -0.141 | | | -0.113 | | | - | |
| *Note:* Lower triangle represents estimated partial contemporaneous correlations, upper triangle represents model-implied marginal contemporaneous correlations | | | | | | | | | | | | | | | |
| **(c) Between-person Relations** | | | | | | | | | | | | | | | |
|  | | 1. | | 2. | | | 3. | | | 4. | | | 5. | | |
| 1. Conduct Problems | | - | | 0.419 | | | 0.676 | | | 0.436 | | | -0.517 | | |
| 2. Emotional Problems | | 0.229 | | - | | | 0.345 | | | 0.521 | | | -0.245 | | |
| 3. Hyperactivity | | 0.520 | | - | | | - | | | 0.462 | | | -0.475 | | |
| 4. Peer Problems | | - | | 0.415 | | | 0.187 | | | - | | | -0.456 | | |
| 5. Prosociality | | -0.276 | | 0.099 | | | -0.133 | | | -0.279 | | | - | | |
| *Note:* Lower triangle represents estimated partial between-person correlations, upper triangle represents model-implied marginal between-person correlations | | | | | | | | | | | | | | | |

| **Table S3**: Childhood Networks – number of times each parameter was included in the case-drop bootstrap  **(a) Temporal Parameters** | | | | | | | | | | | | | | | |
| --- | --- | --- | --- | --- | --- | --- | --- | --- | --- | --- | --- | --- | --- | --- | --- |
|  | | | a. | | | b. | | | c. | | | d. | | | e. |
| 1. Conduct Problems (a) | | | 947 | | | 69 | | | 286 | | | 403 | | | 921 |
| 2. Emotional Problems (b) | | | 277 | | | 947 | | | 584 | | | 357 | | | 46 |
| 3. Hyperactivity (c) | | | 244 | | | 67 | | | 947 | | | 360 | | | 182 |
| 4. Peer Problems (d) | | | 130 | | | 661 | | | 40 | | | 947 | | | 188 |
| 5. Prosociality (e) | | | 444 | | | 61 | | | 57 | | | 108 | | | 947 |
| *Note.* Lower triangle represents directional relations from 1-5 to a-e and the upper triangle directional relations from a-e to 1-5.  **(b) Contemporaneous Parameters** | | | | | | | | | | | | | | | |
|  | 1. | | | | 2. | | | 3. | | | 4. | | | 5. | |
| 1. Conduct Problems | - | | | | 1000 | | | 1000 | | | 1000 | | | 1000 | |
| 2. Emotional Problems | 1000 | | | | - | | | 1000 | | | 1000 | | | 1000 | |
| 3. Hyperactivity | 1000 | | | | 1000 | | | - | | | 884 | | | 1000 | |
| 4. Peer Problems | 1000 | | | | 1000 | | | 884 | | | - | | | 1000 | |
| 5. Prosociality | 1000 | | | | 1000 | | | 1000 | | | 1000 | | | - | |
| **(c) Between-person Parameters** | | | | | | | | | | | | | | | |
|  | | 1. | | 2. | | | 3. | | | 4. | | | 5. | | |
| 1. Conduct Problems | | - | | 1000 | | | 1000 | | | 179 | | | 1000 | | |
| 2. Emotional Problems | | 1000 | | - | | | 79 | | | 1000 | | | 962 | | |
| 3. Hyperactivity | | 1000 | | 79 | | | - | | | 1000 | | | 998 | | |
| 4. Peer Problems | | 179 | | 1000 | | | 1000 | | | - | | | 1000 | | |
| 5. Prosociality | | 1000 | | 962 | | | 998 | | | 1000 | | | - | | |
| *Note:* Each replication (N=1000) was estimated on a subsample including 75% of the data. Results indicate that the contemporaneous and between-person network were very stable across all replications. The temporal network was very stable with respect to autoregressive effects (diagonal) but less stable for cross-domain relations indicating that the network might be less sparse than originally estimated. | | | | | | | | | | | | | | | |

| **Table S4**: Estimates for regularised Adolescence GVAR Model  **(a) Temporal Relations** | | | | | | | | | | | | | | | |
| --- | --- | --- | --- | --- | --- | --- | --- | --- | --- | --- | --- | --- | --- | --- | --- |
|  | | | a. | | | b. | | | c. | | | d. | | | e. |
| 1. Conduct Problems (a) | | | -0.151 | | | - | | | 0.319 | | | - | | | -0.303 |
| 2. Emotional Problems (b) | | | - | | | - | | | - | | | 0.45 | | | - |
| 3. Hyperactivity (c) | | | 0.225 | | | - | | | - | | | - | | | -0.386 |
| 4. Peer Problems (d) | | | - | | | 0.352 | | | - | | | 0.223 | | | - |
| 5. Prosociality (e) | | | -0.180 | | | -0.054 | | | -0.335 | | | - | | | - |
| *Note:* Numeric estimates represent estimated partial directed correlation, diagonal elements represent autocorrelations, lower triangle represents directional relations from 1-5 to a-e and the upper triangle directional relations from a-e to 1-5 (e.g., 0.037 = conduct problems (1) 🡪 peer problems (e); 0.044 = peer problems (5) 🡪 conduct problems (a)). | | | | | | | | | | | | | | | |
| **(b) Contemporaneous Relations** | | | | | | | | | | | | | | | |
|  | 1. | | | | 2. | | | 3. | | | 4. | | | 5. | |
| 1. Conduct Problems | - | | | | 0.221 | | | 0.354 | | | 0.135 | | | -0.339 | |
| 2. Emotional Problems | 0.123 | | | | - | | | 0.235 | | | 0.241 | | | -0.075 | |
| 3. Hyperactivity | 0.301 | | | | 0.169 | | | - | | | 0.083 | | | -0.12 | |
| 4. Peer Problems | 0.078 | | | | 0.215 | | | - | | | - | | | -0.046 | |
| 5. Prosociality | -0.315 | | | | - | | | - | | | - | | | - | |
| *Note:* Lower triangle represents estimated partial contemporaneous correlations, upper triangle represents model-implied marginal contemporaneous correlations | | | | | | | | | | | | | | | |
| **(c) Between-person Relations** | | | | | | | | | | | | | | | |
|  | | 1. | | 2. | | | 3. | | | 4. | | | 5. | | |
| 1. Conduct Problems | | - | | 0.412 | | | 0.591 | | | 0.355 | | | -0.514 | | |
| 2. Emotional Problems | | 0.245 | | - | | | 0.362 | | | 0.468 | | | -0.182 | | |
| 3. Hyperactivity | | 0.429 | | 0.089 | | | - | | | 0.383 | | | -0.372 | | |
| 4. Peer Problems | | - | | 0.369 | | | 0.168 | | | - | | | -0.322 | | |
| 5. Prosociality | | -0.386 | | 0.12 | | | -0.07 | | | -0.188 | | | - | | |
| *Note:* Lower triangle represents estimated partial between-person correlations, upper triangle represents model-implied marginal between-person correlations | | | | | | | | | | | | | | | |

| **Table S5**: Adolescence Networks – number of times each parameter was included in the case-drop bootstrap  **(a) Temporal Parameters** | | | | | | | | | | | | | | | |
| --- | --- | --- | --- | --- | --- | --- | --- | --- | --- | --- | --- | --- | --- | --- | --- |
|  | | | a. | | | b. | | | c. | | | d. | | | e. |
| 1. Conduct Problems (a) | | | 559 | | | 438 | | | 547 | | | 338 | | | 505 |
| 2. Emotional Problems (b) | | | 384 | | | 669 | | | 354 | | | 511 | | | 404 |
| 3. Hyperactivity (c) | | | 416 | | | 494 | | | 536 | | | 357 | | | 546 |
| 4. Peer Problems (d) | | | 301 | | | 498 | | | 329 | | | 769 | | | 333 |
| 5. Prosociality (e) | | | 402 | | | 471 | | | 613 | | | 362 | | | 622 |
| *Note.* Lower triangle represents directional relations from 1-5 to a-e and the upper triangle directional relations from a-e to 1-5.  **(b) Contemporaneous Parameters** | | | | | | | | | | | | | | | |
|  | 1. | | | | 2. | | | 3. | | | 4. | | | 5. | |
| 1. Conduct Problems | - | | | | 841 | | | 891 | | | 312 | | | 945 | |
| 2. Emotional Problems | 841 | | | | - | | | 503 | | | 930 | | | 363 | |
| 3. Hyperactivity | 891 | | | | 503 | | | - | | | 334 | | | 398 | |
| 4. Peer Problems | 312 | | | | 930 | | | 334 | | | - | | | 386 | |
| 5. Prosociality | 945 | | | | 363 | | | 398 | | | 386 | | | - | |
| **(c) Between-person Parameters** | | | | | | | | | | | | | | | |
|  | | 1. | | 2. | | | 3. | | | 4. | | | 5. | | |
| 1. Conduct Problems | | - | | 987 | | | 1000 | | | 178 | | | 1000 | | |
| 2. Emotional Problems | | 987 | | - | | | 647 | | | 1000 | | | 792 | | |
| 3. Hyperactivity | | 1000 | | 647 | | | - | | | 949 | | | 731 | | |
| 4. Peer Problems | | 178 | | 1000 | | | 949 | | | - | | | 854 | | |
| 5. Prosociality | | 1000 | | 792 | | | 731 | | | 854 | | | - | | |
| *Note:* Each replication (N=1000) was estimated on a subsample including 75% of the data. Results indicate that the contemporaneous and between-person network were very stable across all replications. The temporal network was very stable with respect to autoregressive effects (diagonal) but less stable for cross-domain relations indicating that the network might be less sparse than originally estimated. | | | | | | | | | | | | | | | |
